# Correspondence

**Published:** 1875-01

**Authors:** W. W. Allport


### Correspondence,

DR. TAFT:

*My Dear Sir*, Your letter asking for my experience in the use of the "Fluid Lightning" is received.

Several months ago I had a patient, the dentine of whose teeth was so exceedingly sensitive, that it was with great pain to her, and trouble to me, that I could succeed in operating for her, and fill one cavity at a sitting. At about this time I had seen a very severe case of headache cured, in the space of a few moments, by the use of "Fluid Lightning." As the action of this preparation was so prompt, and decided in this case, it occurred to me, that it was possible and *barely* possible, that it might be useful in removing sensitiveness in dentine. I had previously used all the usual remedies for this purpose at hand, except cobalt and arsenous acid, with slight expectations, although hoping that some benefit might be derived from the use of "Fluid Lightning." I procured of Drs. Cram & Melcher a bottle of their preparation, without telling them to what purpose I wished to put it.

When my patient came at her next appointment, she said she felt quite unable to have me operate for her that day, and was sure that the pain would be so great, that she would go home exhausted. She, however, was seated in my chair for the purpose of having some wedges changed between her teeth. I then suggested to her that I should try a new preparation, and see what effect it would have upon the dentine, and it was possible that it might so relieve the sensitiveness, that I could operate. She at last consented that I should make the application. I dried the cavity, and applied the "Lightning," and in a moment or two I proceeded to excavate. After removing a portion of the diseased dentine, she experienced so much pain, that I was obliged to desist from operation. I applied the "Lightning" again, and proceeded to operate as before, she suffering but little, on removing the structure, that had given so much pain a moment before.

Soon, however, in excavating deeper into the cavity, she suffered quite severely. I applied the remedy a third time, and finished preparing the cavity, with but slight suffering, and filled it.

She had suffered so little during this, that I prepared and filled a second cavity at the same sitting, and she assured me that she had suffered far less in having these two cavities prepared and filled, than she ever had in having one prepared and filled at a sitting before. In future operations upon this patient, I used the "Lightning" in preparing cavities, with the same happy results that I have just mentioned, I then tried it in several other cases, and whilst the action in all was not so decided and prompt, as in the case I have just mentioned—but some were equally so—in no case have I used it, without greater or less relief.

At about this time, I found that two biscuspids in my own mouth, on the labial surface near the gum, were beginning to decay, and were exceedingly sensitive. I called upon Dr. Crouse, and he undertook to remove the decay and polish the surfaces. I found the pain so great, that it was difficult for me to allow him to proceed with the operation. I had him apply the "Lightning," and I suffered but little pain, in having the operation finished.

My experience had now become so satisfactory, in the use of this remedy, and wishing others to test it, I requested the proprietors to send samples of it, to dentists in different parts of the country, (yourself among the number), with the simple request, that they should "try it," and report as to its action. In reply, Drs. Cram & Melcher received a good many letters, some speaking of it in the highest terms of commendation, others saying, that in some cases it operated well, whilst in others, but little relief followed its use; none, however, but what speak of it, more or less, favorably.

While in my own hands, I have not found it to operate in all cases with as great satisfaction as in the one I have mentioned, I have never used it without some benefit, and I can truly say, that I have used it with more *general* satisfaction, both to myself and to my patients, than any preparation that

I have ever seen, for the purpose named. From what I have said, I trust that no one will expect that this remedy will at all times, and under all circumstances, operate with uniform results, when used for any *one* purpose. This is not the case with the operation of morphine, quinine, chloroform, ether, or any other remedy, in the *Materia Medica*, peculiar conditions of the system, or constitutional idiosyncrasies have much to do with the action of other remedies, and it can not be expected, that "Fluid Lightning" will be an exception to all others. If, however, dentists will get it, and give it credit for what it really can do, I feel sure, that they will find it so useful, that they will not wish to be without it

It is proper for me to say, that when I first commenced using this preparation, its odor to many people, (and to myself amongst the number), was decidedly objectionable.

At my suggestion, in this respect, that used for dental purposes was changed, and in changing the odor, its efficiency in obtunding the pain of sensitive dentine, has been perceptibly improved, the name *Dental Pain Obtunder* has been given to it.

So many teeth have been ruined by the use of other preparations for destroying sensitiveness in dentine, that the question will very naturally be asked, if the same effects will not follow the use of this remedy. To this, I reply, that so far, I have seen no unpleasant effects follow its use, and from the fact that it can be used without the after irritation or destruction of the mucous membrane, or the most delicate cuticle. I do not apprehend that any unpleasant results will follow its use.

Yours truly,
W. W. ALLPORT.

